# Rational Design and Modification of NphB for Cannabinoids Biosynthesis

**DOI:** 10.3390/molecules29184454

**Published:** 2024-09-19

**Authors:** Wenhao Xia, Shimeng Liu, Huanyu Chu, Xianqing Chen, Lihui Huang, Tao Bai, Xi Jiao, Wen Wang, Huifeng Jiang, Xiao Wang

**Affiliations:** 1 New Cornerstone Science Laboratory, Shaanxi Key Laboratory of Qinling Ecological Intelligent Monitoring and Protection, School of Ecology and Environment, Northwestern Polytechnical University, Xi’an 710072, China; xiawenhao@synbiolab.cn (W.X.); chenxq@synbiolab.cn (X.C.); 2 Jiaxing Synbiolab Technology Co., Ltd., Jiaxing 314000, China; liushimeng@synbiolab.cn (S.L.); huanglihui@synbiolab.cn (L.H.); baitao@synbiolab.cn (T.B.); jiaoxi@synbiolab.cn (X.J.); 3 Key Laboratory of Systems Microbial Biotechnology, Tianjin Institute of Industrial Biotechnology, Chinese Academy of Sciences, Tianjin 300308, China; chuhy@tib.cas.cn

**Keywords:** cannabinoids, NphB, rational design, molecular dynamics

## Abstract

The rapidly growing field of cannabinoid research is gaining recognition for its impact in neuropsychopharmacology and mood regulation. However, prenyltransferase (NphB) (a key enzyme in cannabinoid precursor synthesis) still needs significant improvement in order to be usable in large-scale industrial applications due to low activity and limited product range. By rational design and high-throughput screening, NphB’s catalytic efficiency and product diversity have been markedly enhanced, enabling direct production of a range of cannabinoids, without the need for traditional enzymatic conversions, thus broadening the production scope of cannabinoids, including cannabigerol (CBG), cannabigerolic acid (CBGA), cannabigerovarin (CBGV), and cannabigerovarinic acid (CBGVA). Notably, the W3 mutant achieved a 10.6-fold increase in CBG yield and exhibited a 10.3- and 20.8-fold enhancement in catalytic efficiency for CBGA and CBGV production, respectively. The W4 mutant also displayed an 9.3-fold increase in CBGVA activity. Molecular dynamics simulations revealed that strategic reconfiguration of the active site’s hydrogen bonding network, disulfide bond formation, and enhanced hydrophobic interactions are pivotal for the improved synthetic efficiency of these NphB mutants. Our findings advance the understanding of enzyme optimization for cannabinoid synthesis and lay a foundation for the industrial-scale production of these valuable compounds.

## 1. Introduction

*Cannabis sativa* L. has been utilized for centuries, primarily due to its psychoactive constituents [1]. The main cannabinoids from *Cannabis sativa* L., including cannabigerol (CBG), cannabidiol (CBD), and cannabidiolic acid (CBDA), have shown significant potential in neuropsychopharmacology and mood regulation [2,3,4]. Several countries have approved CBG and CBD as additives in various fields [5,6,7,8,9] including food, beverages, cosmetics, and nutritional supplements [10,11,12]. In recent years, there has been an increase in cannabinoid use in Europe and North America [13,14], leading to more research on *Cannabis sativa* L. secondary metabolites, especially cannabinoid [15,16,17].

Traditional cannabinoid extraction methods from botanical matrices face challenges including the presence of diverse cannabinoid constituents that require complex purification protocols. Moreover, Cannabis sativa L. cultivation involves long growth cycles and extensive land use [18,19]. Consequently, synthetic biology has become an attractive approach to synthesize cannabinoids more efficiently [20,21,22,23]. The prenyltransferase (NphB) is the first step in combining geranyl diphosphate (GPP) and olivetolic acid (oa) to produce cannabigerolic acid (CBGA) [22]. NphB is capable of catalyzing the C- and O-geranylation of a variety of aromatic compounds [24,25]. It can catalyze the geranylation of OA at the C3 position to form CBGA (minor) and at the O2 position to form 2-O-geranylolivetolic acid (2OG, major) [20]. Numerous studies have reported that enhancing its product specificity and kinetic performance enables the efficient synthesis of CBGA [26,27,28]. This is the common precursor of cannabinoid compounds in the cannabis biosynthetic pathway [29,30,31]. CBGA could be further converted into CBDA, THCA, CBD, THC, and other cannabinoids [32,33,34]. Although CBGA biosynthesis and some non-natural analogs have been achieved in yeast- and cell-free catalysis [27,35], the low catalytic activity for NphB still is a critical rate-limiting factor in the synthesis process. There is a strong need for further research into its catalytic mechanisms to improve yields.

In this study, we employed the rational design and modification of NphB to enhance the biosynthesis of various cannabinoid scaffolds. Additionally, we revealed its catalytic mechanism that results in the synthesis of different cannabinoids by NphB mutants. These results lay the foundation for synthesizing rare cannabinoid compounds.

## 2. Results

### 2.1. Screening and Characterization of NphB for CBG Biosynthesis

**Figure 1:**
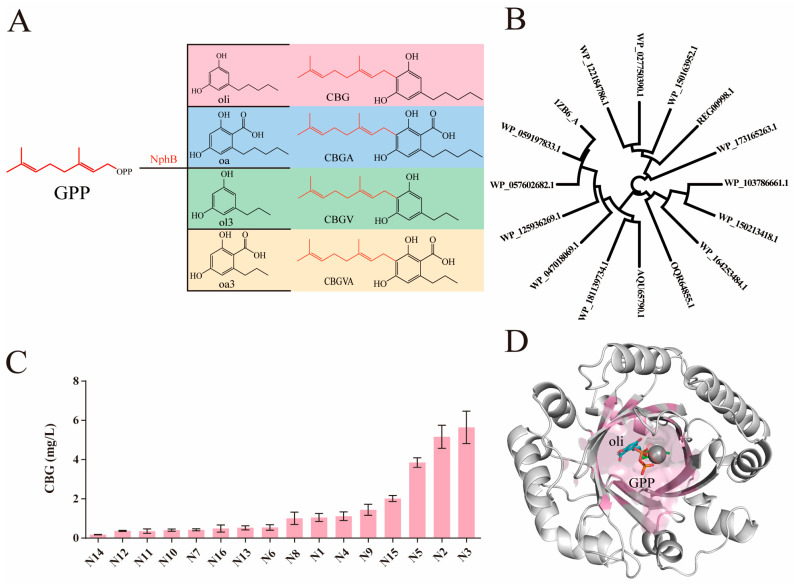
Screening of prenyltransferases orthologous to NphB. (**A**) Yield routes of various cannabinoids by NphB. (**B**) Gene tree for the selected homologous sequences to the previously reported NphB from Streptomyces sp. strain CL190. The GenBank accession: AB187169. (**C**) The CBG yield spectrum of different sequences. The error bars represent standard deviation. Measurements were conducted in triplicate. (**D**) A protein model for the N3 homolog, the pink region delineates the substrate-binding pocket.

To enhance the diversity of products, a comparative study was conducted on the structural differences of various cannabinoids. Rational design led to the selection and use of non-olivetolic acid (non-oa) substrates as alternatives to olivetolic acid (oa) for conjugation with geranyl pyrophosphate (GPP) mediated by NphB. This strategy facilitated the creation of novel cannabinoid scaffolds not derived directly from cannabigerolic acid (CBGA) but synthesized by NphB. These include cannabigerol (CBG) from olivetol (oli), cannabigerovarin (CBGV) from 5-propyl-1,3-benzenediol (ol3), and cannabigerovarinic acid (CBGVA) from 2,4-dihydroxy-6-propylbenzoic acid (oa3) (Figure 1A). The primary objective of this study was to synthesize a spectrum of cannabinoids, with a particular focus on the biosynthesis of CBG, demonstrating the utilization of oli and GPP as precursors in NphB-catalyzed reactions.

To identify potential sequences for our synthetic biology research, the previously reported aromatic prenyltransferase Orf2 from Streptomyces sp. strain CL190 was used as the query sequence to perform a homology search in the NCBI database [25,30]. We selected a total of 100 prenyltransferases sequences with more than 30% identity (Figure S1). Based on the gene tree, we delineated four discrete clades and selected 3 to 5 representative sequences from each clade for further analysis. Finally, a total of 16 prenyltransferases sequences were selected (Figure 1B), followed by a streamlined renaming process for these selected sequences (Table S1).

The efficiency of CBG synthesis from the various sequences was analyzed (Figure 1C), where the sequences N3, N2, and N5 showed relatively high activities, yielding 5.8 mg/L, 5.6 mg/L, and 4.2 mg/L of CBG, respectively. The wild-type enzyme demonstrated peak activity at 30 °C with a 5 mM concentration of magnesium ions in a pH 8.0 buffered solution (Figure S2A–D). To boost the wild-type enzyme’s catalytic activity for CBG, we downloaded the protein structure model of the NphB sequence (1ZB6_A) from the Protein data bank (PDB) database and utilized Rosetta to dock the necessary substrates and cofactors to the active site [36], followed by the optimization of the docked structure (Figure S3) [37,38,39,40]. Our focus was on the residues lining the substrate-binding pocket (Figure 1D), which are critical for the enzyme’s interaction with its substrate. Conformations were deemed plausible based on two criteria: the distance between the substrate reactive site and the α-C of GPP within 5 Å and a negative docking score (binding energy). The most reasonable structure for further analysis was selected based on empirical experience. Superimposition of docking results with the Nphb-GSPP-1,6-DHN complex confirmed the reliability of our findings, illustrating that the oli occupied a similar area within the catalytic pocket, as seen with 1,6-DHN [24,25]. The measured distance from the C5 of oli to the C1 of GPP was measured at 5.1 Å, which is comparable to the 4.2 Å observed in the complex structure of NphB with 1,6-DHN and GSPP. By combining the docking results of the N3 model with substrate interaction and kinetic analysis, we further engineered N3 to improve its catalytic efficiency in producing CBG.

### 2.2. Engineering of NphB for Cannabinoids Biosynthesis

**Figure 2:**
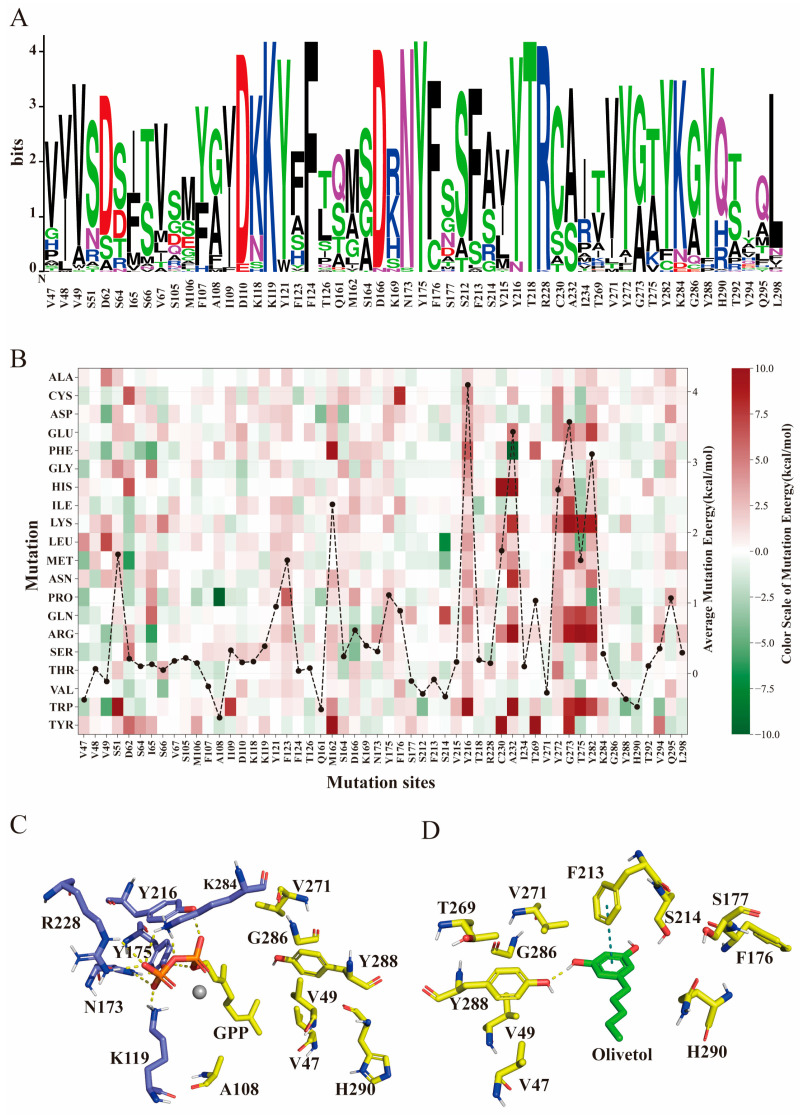
Rational engineering of NphB enzyme. (**A**) Conservation analysis of residues within the NphB active sites. (**B**) Gibbs free energy heatmap of residues within the NphB active sites. The black dots represent each mutation site, and the average change in binding energy after virtual saturation mutagenesis. (**C**) Interaction analysis between geranyl diphosphate (GPP) and surrounding residues. (**D**) Interaction analysis between olivetol and surrounding residues. Yellow dashed lines represent hydrogen bonds, while green dashed lines indicate π-π stacking.

Based on the docking results of the enzyme with the substrates, 54 residues within a 6 Å range of oli and GPP were selected for preliminary analysis as part of the substrate-binding pocket. Conservation analysis led to the exclusion of 10 conserved residues from mutation considerations (Figure 2A). In the reaction between oli and GPP, the pyrophosphate moiety of GPP engages in hydrogen bonding with the side chains of residues K119, N173, Y175, Y216, R228, and K284, thereby securing its position (Figure 2C). The prenyl chain of GPP is embedded in a hydrophobic environment, surrounded by hydrophobic amino acid residues. Olivetol forms hydrogen bonds with Y288 and is stabilized by π-π stacking with the phenyl ring of F213 (Figure 2D). While residues critical for stabilizing GPP’s pyrophosphate group were excluded from the mutagenesis targets to avoid any destabilization of GPP, Y288, and F213, impacting both GPP and olivetol, are still considered viable targets for mutational analysis. To further narrow down the target sites, virtual saturation mutagenesis was performed on the residues within the substrate-binding pocket using Rosetta 2022.08. The change in binding energy for each mutant was used to assess their potential impact on enzyme activity (Figure 2B). A decrease in binding energy indicates a more stable substrate–enzyme complex, which is favorable for catalysis. Consequently, the sites with increased binding energy among all 20 residues were eliminated. Considering the aforementioned criteria, we identified V47, V49, F107, A108, S212, F213, S214, Q161, F176, S177, T269, V271, C286, Y288, and H290 for our mutational experiments.

**Figure 3:**
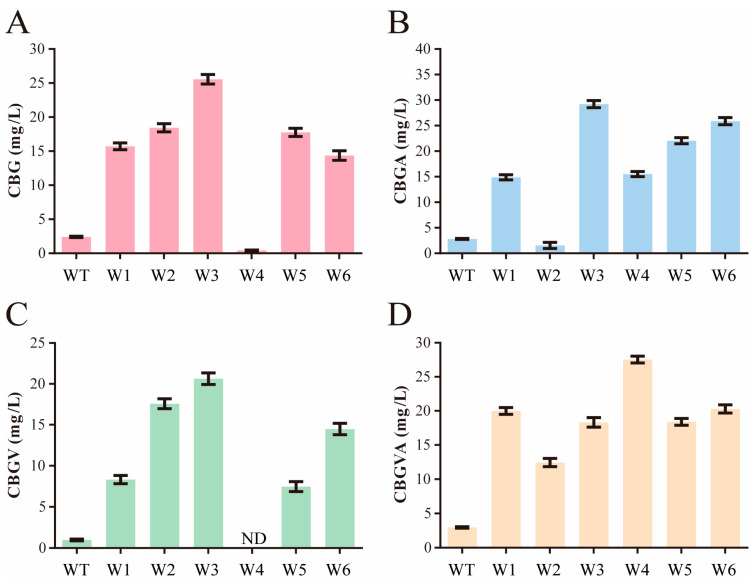
Selection of NphB mutants for the augmented biosynthesis of diverse cannabinoids. (**A**) CBG production by the wild-type and mutant strains. (**B**) CBGA production by the wild-type and mutant strains. (**C**) CBGV production by the wild-type and mutant strains. (**D**) CBGVA production by the wild-type and mutant strains. ND signifies not detected. The error bars represent standard deviation. Measurements were conducted in triplicate. Red represents the synthesis data of CBG, blue represents the synthesis data of CBGA, green represents the synthesis data of CBGV, and yellow represents the synthesis data of CBGVA.

We designed a dual-site combinatorial saturation screening for efficient screening of candidate residues. Considering that the selected mutation residues are either adjacent or very close in spatial position, this basis was used for the dual-site combinatorial saturation screening. We first used a pyrophosphate assay kit for high-throughput preliminary screenings, then cultured and purified clones showing elevated fluorescence values. We then used the purified enzymes to confirm their activity. The first round of screening identified the mutant WT-Q161R/S214H (named W1) as showing the most significant increase in CBG catalytic activity, boosting CBG production from 2.42 mg/L to 16.23 mg/L. Interestingly, the WT-S212A/F213S mutant exhibited good catalytic activity for CBGVA, possibly due to the smaller side chains of the mutated residues allowing the reaction chamber to accommodate larger substrates. Subsequent screenings continued based on W1 and WT-S212A/F213S, focusing on sites with beneficial mutations from the first round. Further mutations based on W1 led to strains W2 (W1-V49C/H290D) and W5 (W1-F176D/S177T/Y288V). Both strains achieved nearly identical CBG yields of approximately 18.62 mg/L. Further mutations based on the W2 strain led to the creation of strain W3 (W2-V47D/G286C), which marked a significant milestone in our research. This strain increased CBG yield to 25.56 mg/L (Figure 3A), a tenfold increase from the original wild-type strain, and exhibited a remarkable 10.3-fold increase in catalytic efficiency for CBGA synthesis (Figure 3B) and a 20.8-fold increase for CBGV production (Figure 3C). Additionally, the mutants W2 and W6 (W2-V47C/G286C) also showed significant improvements, with CBGV production increasing by 17.8 and 14.6 times, respectively. Furthermore, in the biosynthesis of CBGVA through the combination of oa3 and GPP, the W4 (WT-S212A/F213S-Q161R/Y288V) mutant has an 9.3-fold increase in enzyme activity compared to the wild-type (Figure 3D).

### 2.3. Mechanistic Analysis of Production of Diverse Cannabinoids

**Figure 4:**
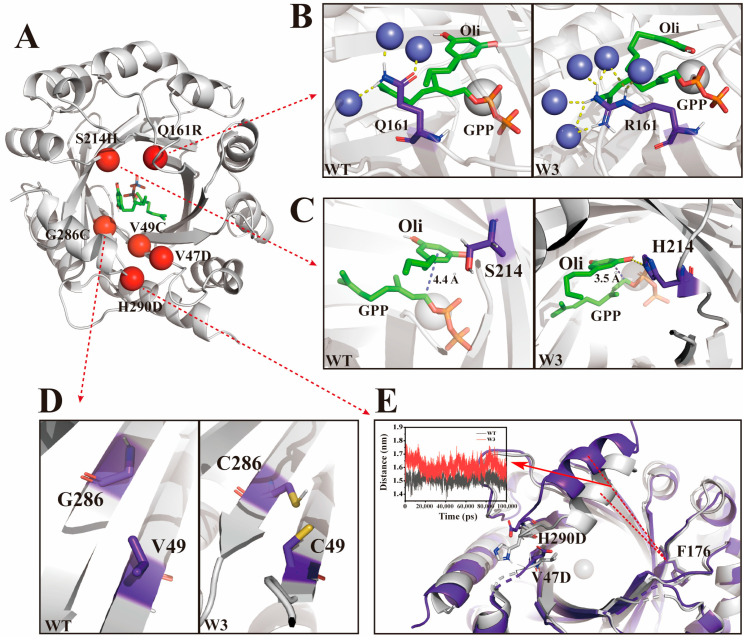
Mechanistic analysis of NphB mutants for diverse cannabinoid scaffolds. (**A**) Overall structural model of NphB, wild-type to W3 point mutations are shown as red dots. (**B**) The mutation of residue 161 results in a change in the quantity of water molecules interacting and the number of hydrogen bonds with these water molecules, where yellow dashed lines denote hydrogen bonds and blue spheres represent water molecules. (**C**) The mutation of residue 214 induces alterations in substrate conformation and a reduction in the reaction distance, yellow dashed lines denote hydrogen bonds, and purple dashed lines signify the reaction distances. (**D**) Residues 286 and 49 are mutated to cysteine, resulting in the formation of a disulfide bond. (**E**) The mutations at positions 47 and 290 increase the flexibility of the α-helix and expand the substrate pocket, where the gray structure denotes the wild-type enzyme, and the purple structure corresponds to the W3 mutant variant. All gray spheres represent metal ions.

To elucidate the molecular basis for the enhanced catalysis of these mutants, we conducted a 100 ns molecular dynamics simulation for both the wild-type and the mutant, then carried out subsequent analysis after confirming the stability of the trajectory based on the root-mean-square deviation (RMSD) (Figure S4). we compared the wild-type NphB with the W3 variant, which showed significantly enhanced activity for four different aromatic substrates. As a promising aromatic prenyltransferase, NphB includes a cylindrical decameric β-barrel filled with solvent at its core, surrounded by α-helices exposed to the solvent. The final helix at the C-terminus forms a lid over the barrel. The W3 variant contains six-point mutations distributed within the β-barrel and at its junction with the lid (Figure 4A). The Q161R mutation has led to an increase in hydrogen bonds with surrounding water molecules from three to seven, and the number of bound water molecules has increased from three in the wild-type to five. This enhancement significantly augments the hydrophobicity within the substrate pocket, thereby stabilizing the hydrophobic segment of the substrate (Figure 4B). The E214H mutation forms a hydrogen bond with the substrate’s phenyl ring hydroxyl group, enhancing substrate stability. Additionally, a conformational change in the substrate occurs, reducing the distance between the GPP C1 and the olivetol C4 from 4.4 Å to 3.5 Å. This substantial reduction in the reactive distance between the two substrates promotes the occurrence of the reaction (Figure 4C). Mutation of residues 286 and 49 to cysteine facilitates the formation of a disulfide bond, which stabilizes the substrate-binding pocket and enhances the overall structural integrity of the enzyme. Moreover, the elevated local hydrophobicity resulting from this mutation is advantageous for the stabilization of the substrate conformation and the carbon cation intermediate (Figure 4D). Residues 47 and 290, situated at the entrance loop of the substrate-binding pocket, regulate substrate entry via conditional α-helix dynamics. In the wild-type enzyme, H290 engages in hydrogen bonding with S46 and V48, thereby limiting the mobility of the α-helix (Figure S5A). In the W3 mutant, the mutations H290D and V47D disrupt the pre-existing hydrogen bond network, leading to the formation of new interactions between D290 and I291 (Figure S5B). The reconfiguration of the hydrogen bond network diminishes the interactions of D290 with neighboring residues, alleviating the restraints imposed on the α-helix. By analyzing the root mean square fluctuation (RMSF) values of WT and mutant W3, the RMSF value of residue F176 in the WT is 0.0376 nm, while in mutant W3 it is 0.044 nm, indicating that the residue has the least fluctuation and its side-chain spatial position is relatively stable (Figure S6). To quantify the motion of the α-helix, we measured its distance to F176. The mutant exhibits an increased distance between the α-helix and F176, which dilates the binding pocket and enhances the α-helix flexibility, allowing for unimpeded substrate interaction (Figure 4E). This enhanced mobility facilitates greater contact between the substrate and GPP, thereby increasing the probability of the catalytic reaction.

**Figure 5:**
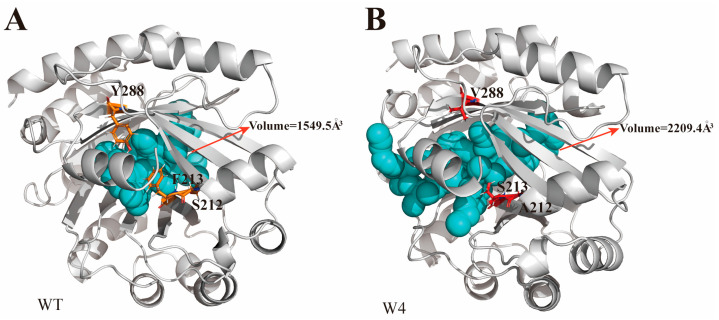
Structural and substrate-binding pocket size comparison between the wild-type (**A**) and the W4 mutant (**B**) variant.

During the screening process, a special mutant W4 was identified, which only had four mutations (S212A, F213S, Q161R and Y288V). Compared to the wild-type, its activity for CBG and CBGV has almost vanished. However, the synthetic efficiency for CBGA and CBGVA has significantly increased, with CBGVA showing the highest activity. W4 exhibits superior catalytic efficiency for substrates with carboxylic groups. Structural analysis reveals that residues S212A, F213S, and Y288V are located near the aromatic ring of the substrate. These mutations introduce smaller side chains compared to the wild-type, creating additional space within the β-barrel. The substrate-binding pocket volume expanded from 1549.5 Å^3^ in the wild-type to 2209.4 Å^3^ in the W4 variant (Figure 5A,B). This expanded binding pocket better accommodates substrates with larger carboxyl groups, enhancing the forward reaction efficiency.

## 3. Discussion

Using computational simulations, we refined NphB to accommodate various substrates, substantially increasing yields of different cannabinoids through one-step catalysis by NphB. Previous studies have focused on enhancing the product specificity and kinetic performance of NphB to increase the yield of CBGA [21,41]. Mutants such as Y288A_G286S or Y288V_A232S dramatically improved NphB kinetics; such point mutations also rendered almost exclusive production of CBGA, leading to 21-fold increase in CBGA titer (744 mg/L) [26,28]. Using a continuous reaction setup in a cell-free system also increased the CBGA titer to 1.25 g/L [27]. Furthermore, the product specificity for CBGA was enhanced by the Q295F mutation, which increased the in vivo activity in E. coli by 20-fold [42]. Building on this work, we conducted detailed mutagenesis and screening of critical NphB enzyme sites. We identified several key amino acid residues that are pivotal for the enzyme’s activity and specificity. These residues include Q161, S214, H290, G286, S212, and F213. By introducing mutations at these sites, either individually or in various combinations, we have achieved differentiated enhancements in the production of several cannabinoids, including CBG, CBGA, CBGVA, and CBGV. Each mutation or combination of mutations was strategically chosen based on their potential to alter the enzyme’s spatial configuration or its dynamic interaction with specific substrates. The analysis of the catalytic mechanisms of different NphB mutants lays a solid foundation for future enhancements of the NphB enzyme. Our results provide novel insights into enzyme catalytic mechanisms and lay the groundwork for identifying even higher-activity variants in the future. Based on the reaction mechanism, mutations that stabilize substrate orientation, stabilize the intermediate, and promote substrate entry and exit can influence the enzyme’s activity from different perspectives.

Cannabinoids continue to be investigated for their prospective therapeutic utilities, with over 113 distinct scaffolds identified thus far [43]. While CBG has attracted substantial attention, other cannabinoids also demonstrate promising medicinal attributes. As additional cannabinoids emerge, innovative bioproduction platforms become imperative. Traditionally, the biosynthetic pathway for cannabinoids involves the catalysis of olivetol and GPP by the enzyme NphB to produce CBGA. Subsequently, using CBGA as a substrate, other cannabinoids such as THCA, CBDA, and CBCA can be synthesized under the catalysis of other enzymes. We developed an innovative approach leveraging NphB’s capabilities in response to the growing diversity of cannabinoids and their potential applications. We have engineered NphB to perform a one-step catalysis that directly produces various cannabinoids, bypassing the need for subsequent enzymatic transformations typically required in traditional pathways. This streamlined approach not only significantly enhances the efficiency of cannabinoid synthesis but also improves carbon utilization within the biosynthetic pathways.

By reducing the enzymatic steps involved, we minimize the metabolic burden on the host systems and enhance the overall yield and purity of the desired cannabinoids. Our approach has fundamentally enhanced the efficiency of cannabinoid synthesis and improved carbon utilization within the biosynthetic pathways. There is potential to transplant these modified NphB enzymes into living systems or incorporate them into multi-enzyme, cell-free configurations for the industrial production of cannabinoids from readily available precursors.

## 4. Materials and Methods

### 4.1. Genes and Reagents

Genes were synthesized by Wuhan Genecreat Biological Engineering Co., Ltd. (Wuhan, China) and primer synthesis was completed by Anshengda Biotechnology Co., Ltd. (Tianjing, China). Mevalonate (MVA), cannabigerol (CBG), and olivetolic acid (oa) were sourced from Sigma-Aldrich (Shanghai, China). Other cannabinoid substrates and analytical standards were provided by Macklin (Shanghai, China). BL21(DE3) chemically competent *E. coli* was purchased from Beijing TransGen Biotech Co., Ltd. (Beijing, China).

### 4.2. Evolution of NphB Enzyme Family

To identify homologous sequences, we used the aromatic prenyltransferase Orf2 from Streptomyces sp. strain CL190 as a query template in the National Center for Biotechnology Information (NCBI) Protein Data Bank, retrieving 100 sequences. Multiple sequence alignment was performed with ClustalW, followed by Gblock extraction of conserved regions (Figure S7). Phylogenetic analysis was then conducted in MEGA to construct a gene tree, enabling selection of 16 enzyme candidates for subsequent experimentation.

### 4.3. Cloning, Expression and Purification of Enzymes

The genes were synthesized by Wuhan Genecreate Bioengineering Co., Ltd. (Wuhan, China) and subcloned into the pET-28a(+) vector via one-step clone assembly. All plasmids were transformed into *E. coli* BL21(DE3), and a single colony was inoculated into 5 mL LB (with 100 μg/mL kanamycin) and grown overnight (220 rpm, 37 °C). This starter culture was diluted into 800 mL 2xYT (with 100 μg/mL kanamycin) and incubated (220 rpm, 37 °C) until an OD_600_ 0.6–0.8 was attained. Protein expression was then induced by adding isopropyl β-D-1-thiogalactopyranoside (IPTG) to a final concentration of 0.5 mM, followed by incubation at 16 °C for 16–20 h. The induced culture was centrifuged (5500 rpm, 10 min, 4 °C), the supernatant discarded, and the cell pellet resuspended in lysis buffer (50 mM Tris, 150 mM NaCl, pH 8.0). The suspension underwent high-pressure homogenization (800–1000 bar, 4 °C, 3–5 min) and was clarified by centrifugation (8000 rpm, 1 h, 4 °C). The resulting supernatant was subjected to immobilized nickel affinity chromatography, washing with buffer (50 mM Tris pH 8.0, 150 mM NaCl, 20 mM imidazole) to remove impurities, and eluting the target protein with buffer (50 mM Tris pH 8.0, 150 mM NaCl, 200 mM imidazole). The eluate was concentrated and stored at −80 °C.

### 4.4. Catalytic Conditions for CBG Synthesis by NphB Homologous Sequences

To evaluate the catalytic proficiency of NphB homologs, reactions were initiated by combining enzymes and cofactors in 50 μL volumes, with final concentrations of 0.5 mM GPP, 1 mM olivetol, 5 mM MgCl_2_, and 50 mM Tris (pH 8.0), and 1.0 mg/mL NphB. Following 8 h incubation at 30 °C, reactions were terminated via addition of 100 μL ethyl acetate, shaking (5–10 s), and centrifuged (12,000× *g*, 3 min) to recover the organic fraction in fresh tubes. The aqueous fraction underwent identical ethyl acetate extraction and pooling of the combined organic layers, drying under vacuum centrifugation, and reconstituting in 50 μL methanol for high performance liquid chromatography (HPLC) analysis. By employing the method of controlled variables, the optimal metal ions, optimal metal ion concentration, optimal temperature, and optimal pH for NphB were tested.

### 4.5. Modification of Prenyltransferase NphB

To probe olivetol binding and its impacts on NphB activity, in silico molecular docking studies were undertaken utilizing Rosetta Ligand Dock. The crystal structure of NphB complexed with substrate (PDB ID: 1ZB6) was obtained from the RCSB Protein Data Bank. The native ligands GSPP and 1,6-dihydroxynaphtalene were removed, leaving the position of the metal ions intact. Using Rosetta, GPP and olivetol were docked into the catalytic center. Rosetta relax was then used to generate 200 models and the lowest energy structure was selected for further docking to optimize the complex (Figure S8). The olivetol substrate structure was downloaded from Zinc and prepared via Schrödinger conformational searches, furnishing a sdf file. The Rosetta molfile_to_params script enabled sdf to params file format conversion alongside the generation of a multi-constant image file. Following ligand and receptor processing, docking was executed with the Rosetta (rosetta_scripts.mpi.linuxgccrelease) application utilizing StartFrom and Transform protocols and 10,000 models. Resulting complexes were ranked by total score and binding energy, with the top 10% visually inspected to eliminate unreasonable poses. The lowest energy binding mode was selected as the final docked complex. Subsequent point mutations employed Rosetta Cartesian_ddG calculations, initially performing two rounds of relax on the complex (200 models each) and utilizing the lowest energy structure for further refinement of all residues within 5 Å of the substrate. The refined complex then underwent saturation mutagenesis of these proximal residues to all other 19 amino acids, evaluating the change in binding energy. Finally, mutants exhibiting both favorable binding energy changes and large magnitude effects were experimentally validated.

### 4.6. Screening of Prenyltransferase Saturated Mutant Library

To screen the initial NphB mutant library, we designed double-site combinatorial saturation mutagenesis primers based on the selected mutation target sites, considering that the chosen mutated residues are adjacent or in close spatial proximity. Variants were constructed using these primers and then transformed into BL21(DE3). Single colonies were inoculated into 96-well plates containing 1 mL LB medium per well supplemented with 100 μg/mL kanamycin and grown overnight (37 °C, 750 rpm). The next day, cultures were diluted to 1% into fresh 96-well plates (1 mL 2YT medium plus 100 μg/mL kanamycin per well) and incubated (37 °C, 750 rpm) until an OD_600_ 0.4–0.6. Protein expression was induced by adding 0.5 mM IPTG followed by 14–16 h at 16 °C. After centrifugation (3500 rpm, 5 min) to harvest cells, pellets were washed in buffer (50 mM Tris pH 8.0, 150 mM NaCl), lysed, and 30 μL magnetic beads were added per well to immobilize proteins. Beads were washed in buffer before adding the reaction components (0.5 mM olivetol, 0.25 mM geranyl pyrophosphate (GPP), 5 mM MgCl_2_, 50 mM Tris pH 8.0, 40 μL final volume) and incubating at 30 °C for 2 h. Preliminary screening relied on a pyrophosphate fluorescence kit, measuring the catalytic activity of the mutants in the initial screening by detecting the fluorescence value of the residual pyrophosphate groups after the reaction. If no mutants with fluorescence values higher than the wild-type were detected in the initial screening, it was considered that no positive mutations occurred at that site. Variants with increased fluorescence were selected as the focus mutation library for scale-up cultivation. The 800 mL expression and purification of NphB variants were performed as previously described. The enzymatic assay consisted of 0.25 mM GPP, 0.5 mM olivetol, 5 mM MgCl_2_, 50 mM Tris pH 8.0, and 3 mg/mL NphB in a total volume of 40 μL, incubated at 30 °C for 2 h. The reaction was quenched with ethyl acetate, reconstituted in 50 μL methanol, and analyzed by HPLC as previously detailed.

### 4.7. In Vitro Synthesis of CBG, CBGA, CBGV and CBGVA

To evaluate the catalytic proficiency of NphB mutants, reactions were initiated by combining enzymes and cofactors in 50 μL volumes, with final concentrations of 0.25 mM GPP, 0.5 mM olivetol (oa/ol3/oa3), 5 mM MgCl_2_, and 50 mM Tris (pH 8.0), and 1.0 mg/mL NphB. Following 6 h incubation at 30 °C, reactions were terminated via addition of 100 μL ethyl acetate, shaking (5–10 s), and centrifuged (12,000× *g*, 3 min) to recover the organic fraction in fresh tubes. The aqueous fraction underwent identical ethyl acetate extraction and pooling of the combined organic layers, drying under vacuum centrifugation, and reconstituting in 50 μL methanol for high performance liquid chromatography (HPLC) analysis (Figure S9). CBG quantitation relied on reverse phase fractionation over a C18 column (250 mm × 4.6 mm × 5 μm) using an Alliance Waters e2695 HPLC system with 1 mL/min flow rate. Gradient elution employed water + 0.1% formic acid (solvent A) and acetonitrile + 0.1% formic acid (solvent B) mobile phases, holding B at 70% for 1 min followed by a 6 min increase to 77%, holding for 10 min, and re-equilibration to 70% B, totaling 26 min.

### 4.8. Molecular Dynamics

Molecular dynamics simulations were performed using the GRMOMACS 2020.6 package, the protein system used Amber14sb force field, the general amber force field (GAFF) was used for the ligands [44]. The TIP3P water model with an edge of 10 Å was used to solvate the complex system in a cubic box. The charge was neutralized by adding Sodium ions. The long-range electrostatic interactions were treated using the particle mesh Ewald (PME) method with a 1.0 nm cutoff. Minimization was performed by 5000 steps of steepest-descent minimization procedure. Subsequently, the NVT (constant number of atoms, volume, and temperature) and NPT (constant number of atoms, pressure, and temperature) ensemble were used to carry out a 100 ps restricted simulation. Finally, the maximum cluster of structures in the trajectory between 40 and 50 ns of simulations was used for structure analysis. PyMOL was used for structural visualization and structural analysis.

## 5. Conclusions

NphB is often used as a replacement in the prenylation step for producing cannabinoid cannabigerolic acid (CBGA). Our research highlights the untapped potential of NphB in the synthesis of diverse cannabinoids, with a focus on the rarer variants. Through rational design strategies, we have optimized the microenvironment within the NphB substrate-binding pocket, significantly improving the synthesis efficiency of CBG, CBGA, CBGV, and CBGVA. Molecular dynamics simulations have revealed that key factors in enhancing the synthetic capabilities of NphB mutants include the reconfiguration of the active site’s hydrogen bonding network, disulfide bond formation, and augmented hydrophobic interactions. These insights offer valuable guidance for the future refinement of the NphB enzyme and contribute to the broader understanding of protein engineering efforts catalyzed by prenyltransferases.

## Data Availability

The raw data that support this study are available from the corresponding authors upon reasonable request.

## Supplementary Materials

The following supporting information can be downloaded at: https://www.mdpi.com/article/10.3390/molecules29184454/s1, Table S1: Streamlined nomenclature for the 16 selected prenyltransferase sequences. Figure S1: The construction of a gene tree based on the homologous sequences of NphB. Figure S2: Investigating the reaction conditions influencing NphB’s efficiency in CBG synthesis. Figure S3: Protein NphB model construction and substrate docking. Figure S4: The RMSD values of the wild-type (A), mutant W3 (B) and mutant W4 (C). Figure S5: Analysis of the hydrogen bond network at positions 47 and 290 in both the wild-type and W3 mutant strains. Figure S6: The RMSF values of the wild-type (A) and mutant W3 (B). Figure S7: Alignment of amino acid sequences of NphB with its homologous sequences. Figure S8. The predicted binding free energies by molecular docking oa/oa3/oli/ol3 into WT-NphB. Figure S9. High-performance liquid phase detection of CBG, CBGA, CBGV and CBGVA.

## References

[1] Wrobel T., Dreger M., Wielgus K., Slomski R. (2018). The application of plant in vitro cultures in cannabinoid production. Biotechnol. Lett..

[2] Pertwee R.G. (2008). The diverse CB1 and CB2 receptor pharmacology of three plant cannabinoids: Delta9-tetrahydrocannabinol, cannabidiol and delta9-tetrahydrocannabivarin. Br. J. Pharmacol..

[3] Cascio M.G., Gauson L.A., Stevenson L.A., Ross R.A., Pertwee R.G. (2010). Evidence that the plant cannabinoid cannabigerol is a highly potent alpha2-adrenoceptor agonist and moderately potent 5HT1A receptor antagonist. Br. J. Pharmacol..

[4] De Petrocellis L., Di Marzo V. (2010). Non-CB1, non-CB2 receptors for endocannabinoids, plant cannabinoids, and synthetic cannabimimetics: Focus on G-protein-coupled receptors and transient receptor potential channels. J. Neuroimmune Pharmacol..

[5] Kogan N.M., Lavi Y., Topping L.M., Williams R.O., McCann F.E., Yekhtin Z., Feldmann M., Gallily R., Mechoulam R. (2021). Novel CBG Derivatives Can Reduce Inflammation, Pain and Obesity. Molecules.

[6] Lim K.J.H., Lim Y.P., Hartono Y.D., Go M.K., Fan H., Yew W.S. (2021). Biosynthesis of Nature-Inspired Unnatural Cannabinoids. Molecules.

[7] Nachnani R., Raup-Konsavage W.M., Vrana K.E. (2021). The Pharmacological Case for Cannabigerol. J. Pharmacol. Exp. Ther..

[8] Radwan M.M., Chandra S., Gul S., ElSohly M.A. (2021). Cannabinoids, Phenolics, Terpenes and Alkaloids of Cannabis. Molecules.

[9] Walsh K.B., McKinney A.E., Holmes A.E. (2021). Minor Cannabinoids: Biosynthesis, Molecular Pharmacology and Potential Therapeutic Uses. Front. Pharmacol..

[10] Gulck T., Moller B.L. (2020). Phytocannabinoids: Origins and Biosynthesis. Trends Plant Sci..

[11] Wiles D., Shanbhag B.K., O'Brien M., Doblin M.S., Bacic A., Beddoe T. (2022). Heterologous production of Cannabis sativa-derived specialised metabolites of medicinal significance—Insights into engineering strategies. Phytochemistry.

[12] Perez E., Fernandez J.R., Fitzgerald C., Rouzard K., Tamura M., Savile C. (2022). In Vitro and Clinical Evaluation of Cannabigerol (CBG) Produced via Yeast Biosynthesis: A Cannabinoid with a Broad Range of Anti-Inflammatory and Skin Health-Boosting Properties. Molecules.

[13] Schachtsiek J., Warzecha H., Kayser O., Stehle F. (2018). Current Perspectives on Biotechnological Cannabinoid Production in Plants. Plant. Med..

[14] Melzer R., McCabe P.F., Schilling S. (2022). Evolution, genetics and biochemistry of plant cannabinoid synthesis: A challenge for biotechnology in the years ahead. Curr. Opin. Biotechnol..

[15] Petrocellis L.D., Ligresti A., Moriello A.S., Allarà M., Bisogno T., Petrosino S., Stott C.G., Marzo V.D. (2011). Effects of cannabinoids and cannabinoid-enriched Cannabis extracts on TRP channels and endocannabinoid metabolic enzymes. Br. J. Pharmacol..

[16] Borrelli F., Fasolino I., Romano B., Capasso R., Maiello F., Coppola D., Orlando P., Battista G., Pagano E., Di Marzo V. (2013). Beneficial effect of the non-psychotropic plant cannabinoid cannabigerol on experimental inflammatory bowel disease. Biochem. Pharmacol..

[17] Burstein S. (2015). Cannabidiol (CBD) and its analogs: A review of their effects on inflammation. Bioorganic Med. Chem..

[18] Viskovi J., Zheljazkov V., Sikora V., Noller J., Latkovi D., Ocamb C., Koren A.J.A. (2023). Industrial Hemp (*Cannabis sativa* L.) Agronomy and Utilization: A Review. Agronomy.

[19] Olah A., Markovics A., Szabo-Papp J., Szabo P.T., Stott C., Zouboulis C.C., Biro T. (2016). Differential effectiveness of selected non-psychotropic phytocannabinoids on human sebocyte functions implicates their introduction in dry/seborrhoeic skin and acne treatment. Exp. Dermatol..

[20] Zirpel B., Degenhardt F., Martin C., Kayser O., Stehle F. (2017). Engineering yeasts as platform organisms for cannabinoid biosynthesis. J. Biotechnol..

[21] Qian S., Clomburg J.M., Gonzalez R. (2019). Engineering Escherichia coli as a platform for the in vivo synthesis of prenylated aromatics. Biotechnol. Bioeng..

[22] Blatt-Janmaat K., Qu Y. (2021). The Biochemistry of Phytocannabinoids and Metabolic Engineering of Their Production in Heterologous Systems. Int. J. Mol. Sci..

[23] Pattnaik F., Nanda S., Mohanty S., Dalai A.K., Kumar V., Ponnusamy S.K., Naik S. (2022). Cannabis: Chemistry, extraction and therapeutic applications. Chemosphere.

[24] Kuzuyama T., Noel J., Richard S.J.N. (2005). Structural basis for the promiscuous biosynthetic prenylation of aromatic natural products. Nature.

[25] Kumano T., Richard S.B., Noel J.P., Nishiyama M., Kuzuyama T. (2008). Chemoenzymatic syntheses of prenylated aromatic small molecules using Streptomyces prenyltransferases with relaxed substrate specificities. Bioorganic Med. Chem..

[26] Bowie J.U., Valliere M., Korman T.P., Woodall N. (2020). Biosynthetic Platform for the Production of Cannabinoids and Other Prenylated Compounds. WO Patent.

[27] Valliere M.A., Korman T.P., Arbing M.A., Bowie J.U. (2020). A bio-inspired cell-free system for cannabinoid production from inexpensive inputs. Nat. Chem. Biol..

[28] Valliere M.A., Korman T.P., Woodall N.B., Khitrov G.A., Taylor R.E., Baker D., Bowie J.U. (2019). A cell-free platform for the prenylation of natural products and application to cannabinoid production. Nat. Commun..

[29] Bonitz T., Alva V., Saleh O., Lupas A.N., Heide L. (2011). Evolutionary relationships of microbial aromatic prenyltransferases. PLoS ONE.

[30] Yang Y., Miao Y., Wang B., Cui G., Merz K.M. (2012). Catalytic mechanism of aromatic prenylation by NphB. Biochemistry.

[31] Johnson B.P., Scull E.M., Dimas D.A., Bavineni T., Bandari C., Batchev A.L., Gardner E.D., Nimmo S.L., Singh S. (2020). Acceptor substrate determines donor specificity of an aromatic prenyltransferase: Expanding the biocatalytic potential of NphB. Appl. Microbiol. Biotechnol..

[32] Carvalho A., Hansen E.H., Kayser O., Carlsen S., Stehle F. (2017). Designing microorganisms for heterologous biosynthesis of cannabinoids. FEMS Yeast Res..

[33] Li H., Liu Y., Tian D., Tian L., Ju X., Qi L., Wang Y., Liang C. (2020). Overview of cannabidiol (CBD) and its analogues: Structures, biological activities, and neuroprotective mechanisms in epilepsy and Alzheimer's disease. Eur. J. Med. Chem..

[34] McRae G., Melanson J.E. (2020). Quantitative determination and validation of 17 cannabinoids in cannabis and hemp using liquid chromatography-tandem mass spectrometry. Anal. Bioanal. Chem..

[35] Luo X., Reiter M.A., d'Espaux L., Wong J., Denby C.M., Lechner A., Zhang Y., Grzybowski A.T., Harth S., Lin W. (2019). Complete biosynthesis of cannabinoids and their unnatural analogues in yeast. Nature.

[36] Alford R.F., Leaver-Fay A., Jeliazkov J.R., O'Meara M.J., DiMaio F.P., Park H., Shapovalov M.V., Renfrew P.D., Mulligan V.K., Kappel K. (2017). The Rosetta All-Atom Energy Function for Macromolecular Modeling and Design. J. Chem. Theory Comput..

[37] Chaudhury S., Berrondo M., Weitzner B.D., Muthu P., Bergman H., Gray J.J. (2011). Benchmarking and analysis of protein docking performance in Rosetta v3.2. PLoS ONE.

[38] Weitzner B.D., Jeliazkov J.R., Lyskov S., Marze N., Kuroda D., Frick R., Adolf-Bryfogle J., Biswas N., Dunbrack R.L., Gray J.J. (2017). Modeling and docking of antibody structures with Rosetta. Nat. Protoc..

[39] Cramer P.J.N.S., Biology M. (2021). AlphaFold2 and the future of structural biology. Nat. Struct. Mol. Biol..

[40] Bryant P., Pozzati G., Elofsson A. (2022). Improved prediction of protein-protein interactions using AlphaFold2. Nat. Commun..

[41] Lim K.J.H., Hartono Y.D., Xue B., Go M.K., Fan H., Yew W.S. (2022). Structure-Guided Engineering of Prenyltransferase NphB for High-Yield and Regioselective Cannabinoid Production. ACS Catal..

[42] Kayser O., Stehle F.O. (2020). Biotechnological Production of Cannabinoids. WO Patent.

[43] Alves V.L., Goncalves J.L., Aguiar J., Teixeira H.M., Camara J.S. (2020). The synthetic cannabinoids phenomenon: From structure to toxicological properties. A review. Crit. Rev. Toxicol..

[44] Abraham M.J., Murtola T., Schulz R., Páll S., Smith J.C., Hess B., Lindahl E. (2015). GROMACS: High performance molecular simulations through multi-level parallelism from laptops to supercomputers. SoftwareX.

