# Supplementary material for: Rational Design and Modification of NphB for Cannabinoids Biosynthesis
Source: Molecules. 2024 Sep 19;29(18):4454. doi: 10.3390/molecules29184454 (PMC11434003; doi:10.3390/molecules29184454)
Supplement: Supplementary file 1 [file molecules-29-04454-s001.zip › molecules-3188338-supplementary.pdf]

Supplementary materials

Rational Design and Modification of NphB for Cannabinoids Biosynthesis

Wenhao Xia[1,2,4], Shimeng Liu [2,4], Huanyu Chu[3,4], Xianqing Chen[1,2], Lihui Huang[2], Tao Bai[2], Xi Jiao[2], Wen Wang[1,*], Huifeng Jiang[3,*], Xiao Wang[2,*]

1 New Cornerstone Science Laboratory, Shaanxi Key Laboratory of Qinling Ecological Intelligent Monitoring and Protection, School of Ecology and Environment, Northwestern Polytechnical University, Xi'an 710072, China; Xiawenhao@synbiolab.cn (W.X.); chenxq@synbiolab.cn (X.C.);

2 Jiaxing Synbiolab Technology Co., Ltd., Jiaxing 314000, China; liushimeng@synbiolab.cn (S.L.); huanglihui@synbiolab.cn (L.H.); baitao@synbiolab.cn (T.B.); jiaoxi@synbiolab.cn (X.J.);

3 Key Laboratory of Systems Microbial Biotechnology, Tianjin Institute of Industrial Biotechnology, Chinese Academy of Sciences, Tianjin 300308, China;
chuhy@tib.cas.cn (H.C.);

4 These authors contributed equally: Wenhao Xia, Shimeng Liu, Huanyu Chu

* Correspondence: wangxiao@synbiolab.cn (X.W.); jiang_hf@tib.cas.cn (H.J.); wenwang@nwpu.edu.cn (W.W.); Tel.: +86-150-8708-7320 (X.W.); +86-136-2211-0805 (H.J.); +86-186-2941-8666 (W.W.);

Supplementary Tables

Table S1: Streamlined nomenclature for the 16 selected prenyltransferase sequences.

Simple name	Locus	Source
N1	WP_057602682.1	*Streptomyces_sp._Root1310*
N2	WP_059197833.1	*Streptomyces_antibioticus*
N3	1ZB6_A	*Streptomyces_sp.*
N4	WP_122184786.1	*Streptomyces_triticirhizae*
N5	WP_047018069.1	*unclassified_Streptomyces*
N6	WP_125936269.1	*Streptomyces_sp._WAC_06738*
N7	AQU65790.1	*Streptomyces_niveus*
N8	WP_150163952.1	*Streptomyces*
N9	WP_181139734.1	*Streptomyces_sp._Ru71*
N10	WP_173165263.1	*Phytohabitans_suffuscus*
N11	WP_103786661.1	*Streptomyces_sp._Ru71*
N12	WP_164253484.1	*Streptomyces_sp._S4.7*
N13	OQR64855.1	*Streptomyces_sp._B9173*
N14	WP_150213418.1	*Streptomyces_venezuelae*
N15	WP_027750390.1	*Streptomyces_sp._CNH287*
N16	REG00998.1	*Asanoa ferruginea*

Supplementary Figures

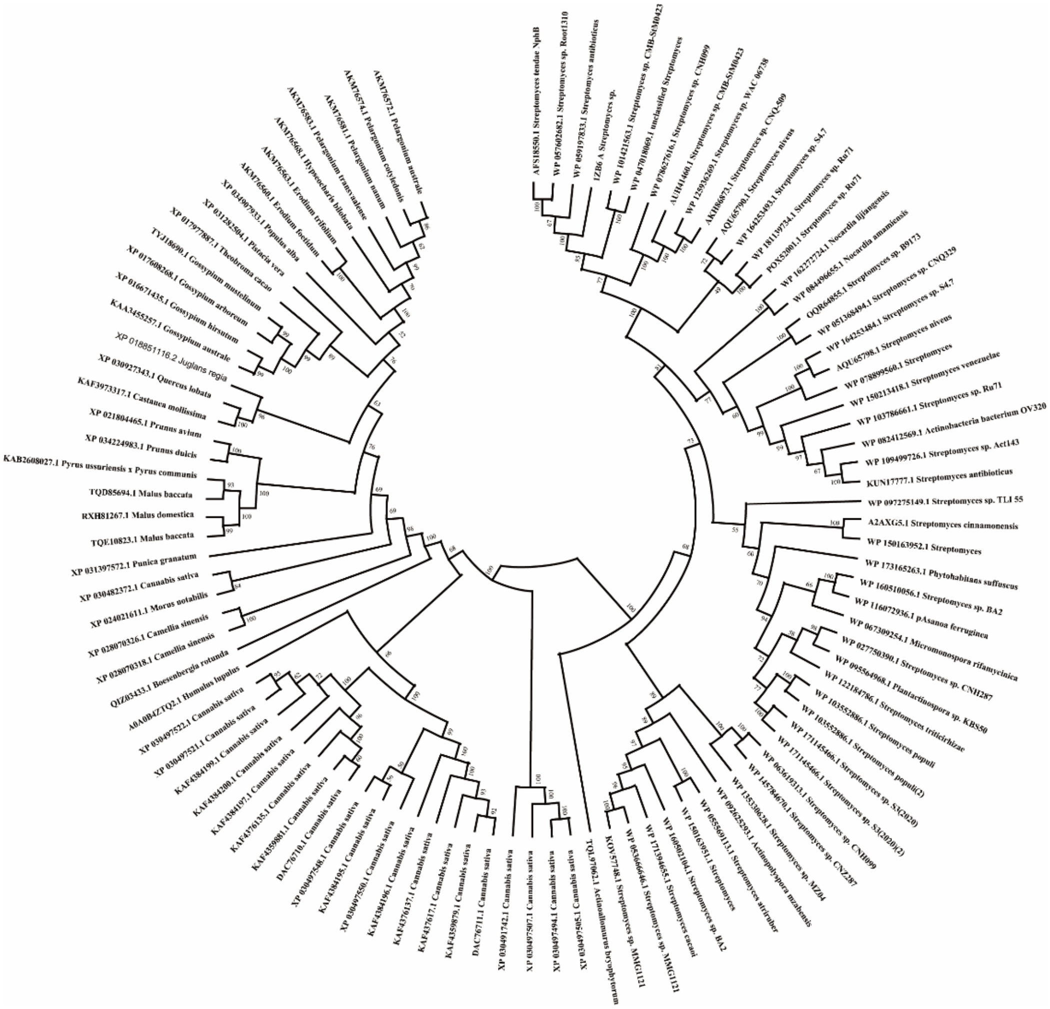

Figure S1: The construction of a gene tree based on the homologous sequences of NphB.

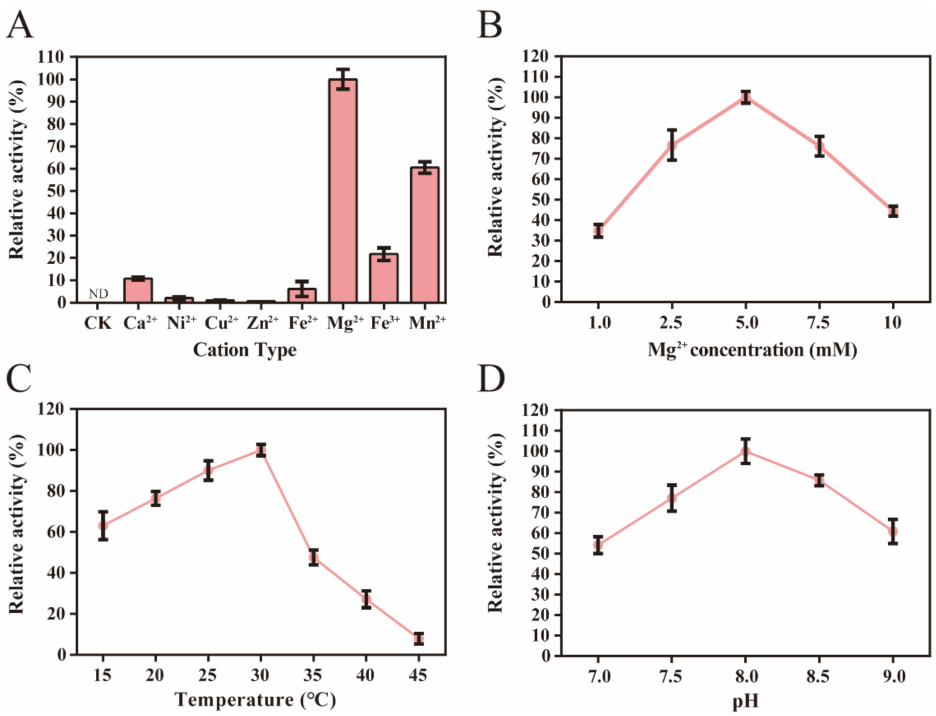

Figure S2: Investigating the reaction conditions influencing NphB's efficiency in CBG synthesis. (**A**) The effect of diverse cation types on enzymatic activity, using the activity in the control without added metal ions (CK) as a baseline, with magnesium ion catalytic activity normalized to 100%, and ND signifies 'not detected'. (**B**) Evaluation of the optimal magnesium ion concentration for the reaction. (**C**) Determination of the most favorable temperature for the reaction. (**D**) Assessment of the ideal pH for the reaction. Error bars indicate standard deviation; experiments performed in triplicate.

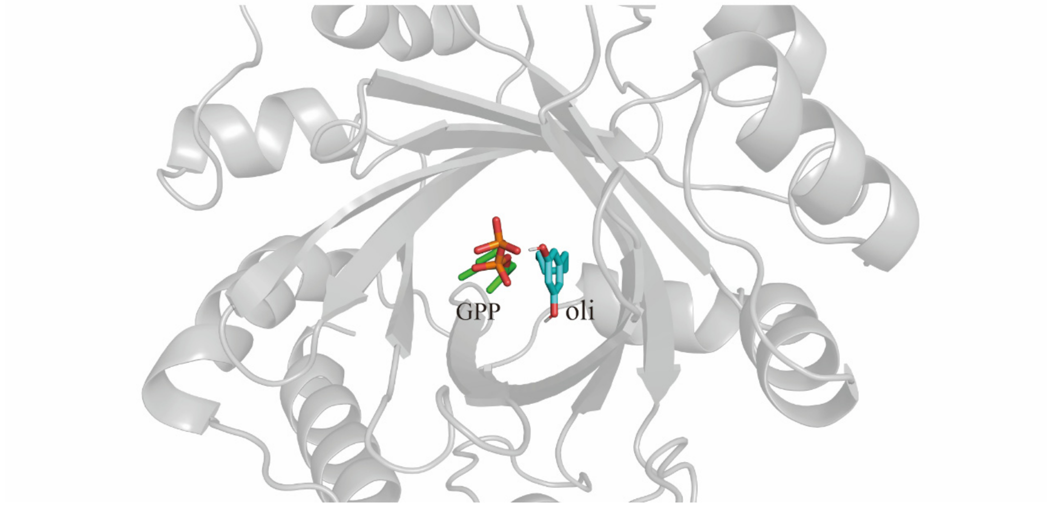

Figure S3: Protein NphB model construction and substrate docking

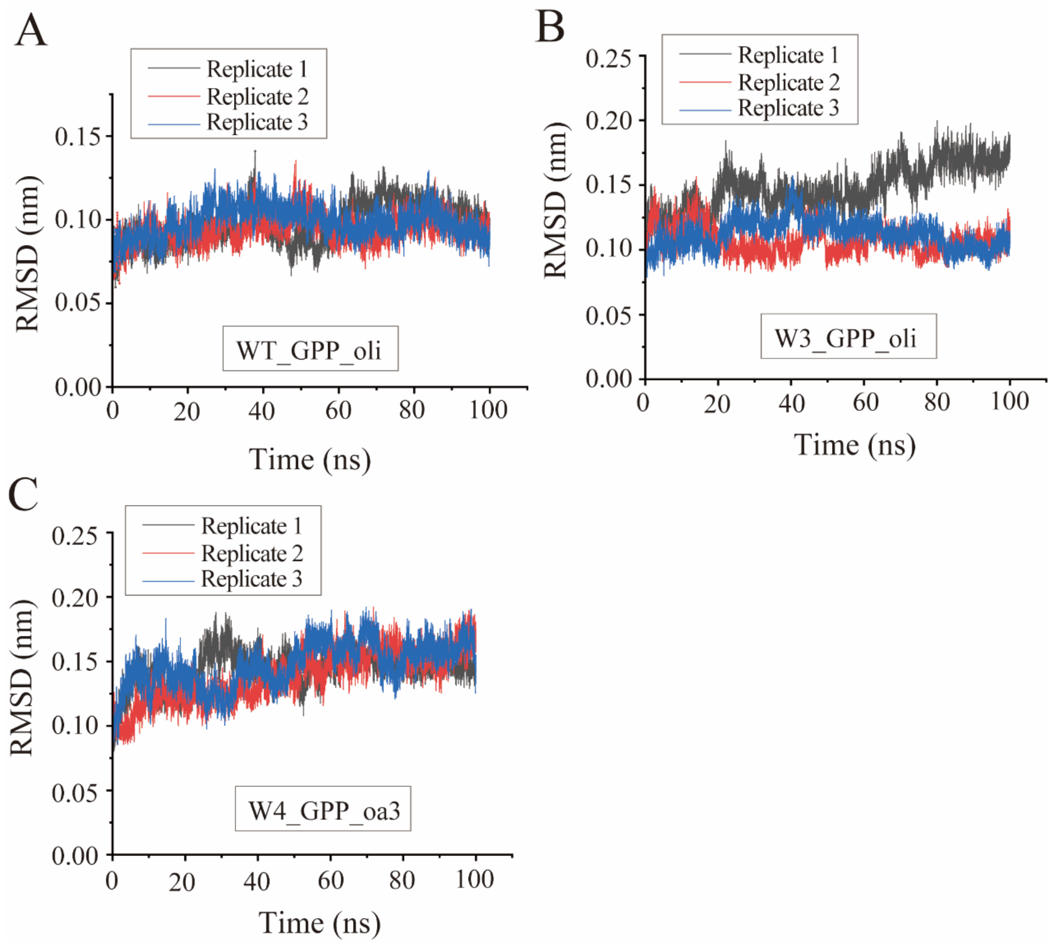

Figure S4: The RMSD values of the wild-type (A), mutant W3 (B) and mutant W4 (C).

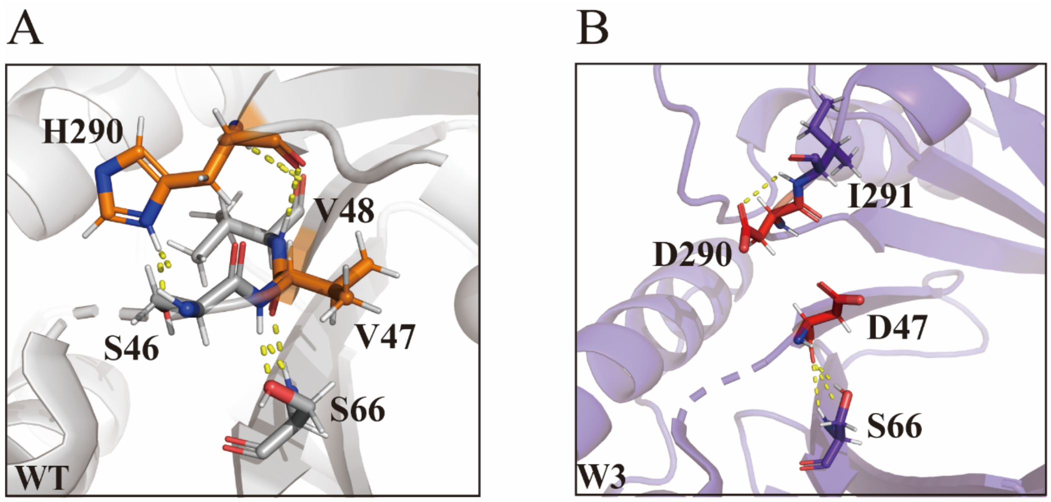

Figure S5: Analysis of the hydrogen bond network at positions 47 and 290 in both the wild-type and W3 mutant strains. (**A**) In the wild-type enzyme, H290 engages in hydrogen bonding with S46 and V48, while V47 is hydrogen-bonded to S66, resulting in a total of eight hydrogen bond pairs. (**B**) In the W3 mutant, D290 forms a single hydrogen bond with I291, and V47 retains its hydrogen bond with S66, resulting in a total of four hydrogen bond pairs. The H290D mutation in the mutant diminishes the interactions of D290 with neighboring residues, easing the restrictions on the alpha-helix.

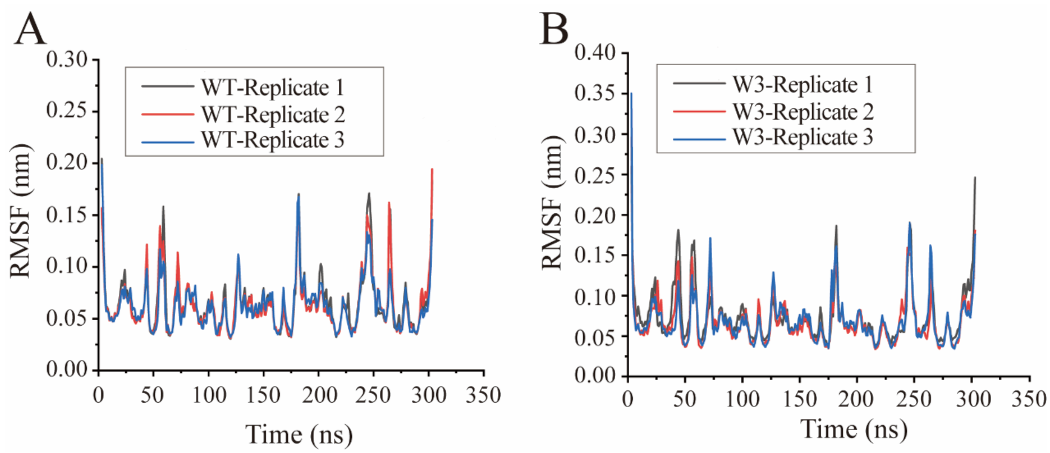

Figure S6: The RMSF values of the wild-type (A) and mutant W3 (B). Residue F176 is located on the beta-sheet within the TIM barrel. Analysis of the protein RMSF values shows that the RMSF value for F176 in the wild-type protein is 0.0376 nm, and in the mutant W3, it is 0.044 nm, indicating minimal fluctuation of the residue and a relatively stable side chain spatial position.

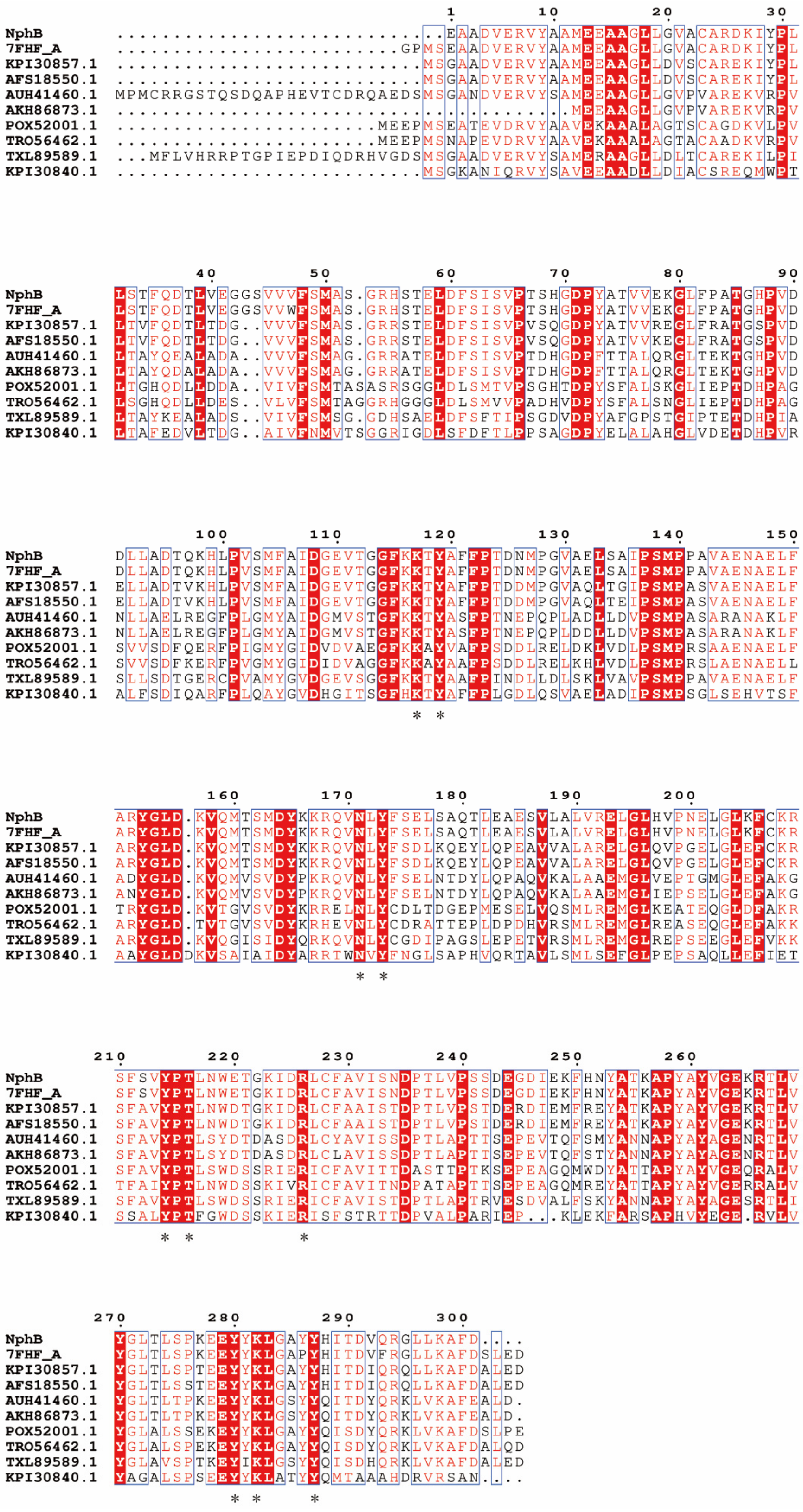

Figure S7: Alignment of amino acid sequences of NphB with its homologous sequences.

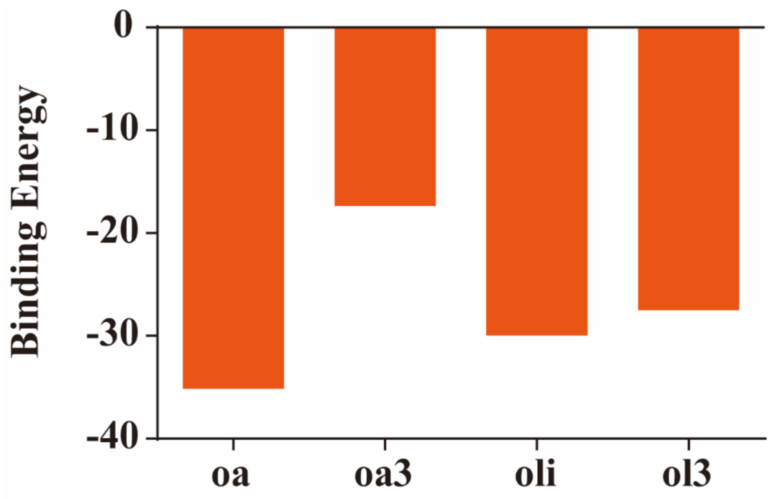

Figure S8. The predicted binding free energies by molecular docking oa/oa3/oli/ol3 into WT-NphB.

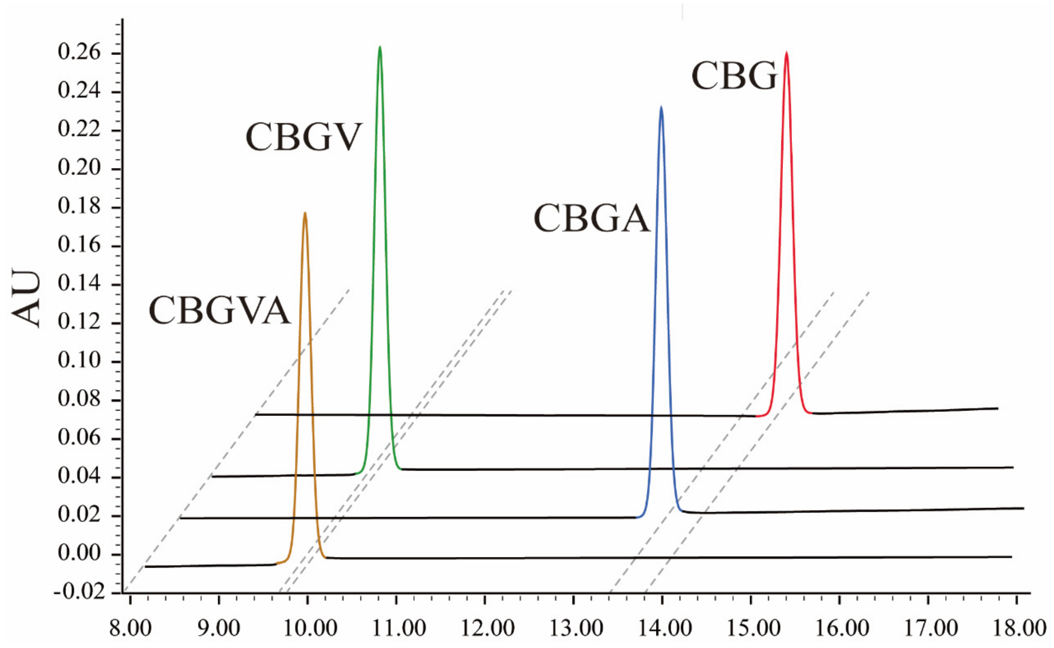

Figure S9. High-performance liquid phase detection of CBG, CBGA, CBGV and CBGVA.
